# Non-linear correlation between amylase day 2 to day 1 ratio and incidence of severe acute pancreatitis

**DOI:** 10.3389/fcimb.2022.910760

**Published:** 2022-11-22

**Authors:** Wandong Hong, Luyao Zheng, Yajing Lu, Minhao Qiu, Ye Yan, Zarrin Basharat, Maddalena Zippi, Vincent Zimmer, Wujun Geng

**Affiliations:** ^1^ Department of Gastroenterology and Hepatology, the First Affiliated Hospital of Wenzhou Medical University, Wenzhou, Zhejiang, China; ^2^ Department of Ultrasonography, the First Affiliated Hospital of Wenzhou Medical University, Wenzhou, Zhejiang, China; ^3^ Jamil-ur-Rahman Center for Genome Research, Dr. Panjwani Centre for Molecular Medicine and Drug Research, International Center for Chemical and Biological Sciences, University of Karachi, Karachi, Pakistan; ^4^ Unit of Gastroenterology and Digestive Endoscopy, Sandro Pertini Hospital, Rome, Italy; ^5^ Department of Medicine II, Saarland University Medical Center, Saarland University, Homburg, Germany; ^6^ Department of Medicine, Marienhausklinik St. Josef Kohlhof, Neunkirchen, Germany; ^7^ Department of Anesthesiology, the First Affiliated Hospital of Wenzhou Medical University, Wenzhou, Zhejiang, China

**Keywords:** amylases, early diagnosis, risk factors, acute pancreatitis, severe acute pancreatitis

## Abstract

**Background:**

This study aimed to assess whether the amylase day 2/amylase day 1 ratio was associated with severe acute pancreatitis (SAP).

**Methods:**

We retrospectively enrolled 464 patients with acute pancreatitis. Serum amylase was measured on admission (day 1) and 24 h later (day 2). Univariable logistic regression with restricted cubic spline analysis, multivariable logistic analysis, and receiver operating characteristic curve analysis was used to evaluate the relationship between the amylase day 2/amylase day 1 ratio and SAP.

**Results:**

A non-linear association between the amylase day 2/amylase day 1 ratio and SAP was observed. The multivariable logistic analysis confirmed that a high amylase day 2/amylase day 1 ratio (≥0.3) was independently associated with the development of SAP (OR: 6.62). The area under the receiver operating characteristic curve (AUC) of the amylase day 2/amylase day 1 ratio, as a predictive factor for SAP, was 0.65. When amylase ratio ≥0.3 was counted as 1 point and added to the BISAP score to build a new model named the BISAPA (BISAP plus Amylase ratio) score (AUC = 0.86), it improved the diagnostic power of the original BISAP score (AUC = 0.83) for SAP. With a cut-off value of 3, the BISAPA score achieved a sensitivity of 66.0%, a specificity of 86.7%, and diagnostic accuracy of 84.48%.

**Conclusions:**

There is a non-linear correlation between the amylase day 2/amylase day 1 ratio and the incidence of SAP. BISAPA score might also be a useful tool for the same purpose.

## Highlights

① There is a nonlinear relationship between the amylase day 2/amylase day 1 ratio and the incidence of SAP.

② With a cut-off value of 0.3, amylase ratio achieved a sensitivity of 92.0% and a specificity of 33.8% for the prediction of SAP.

③ The advantages of amylase estimation are its technical simplicity, easy availability, and high sensitivity.

④ When amylase ratio ≥0.3 is counted as 1 point and added to the BISAP score, it significantly improves diagnostic power compared to the original BISAP score (AUC, 0.86 versus 0.83).

## Introduction

Acute pancreatitis (AP) is a common gastrointestinal disorder with marked variation in severity. In most patients, AP has a self-limiting and mild course. However, a subset of 10%–20% of patients might progress to SAP with high mortality (Hong et al., 2019).

Early identification of high-risk patients on admission may help physicians to stratify the patients who would benefit the most from close surveillance or aggressive intervention (2). Early risk assessment of patients with AP through reliable methods is necessary to potentially improve the clinical outcome, while reduce the treatment cost and length of hospitalization. A great deal of effort has focused on the development of approaches for early risk stratification of AP. However, existing scoring systems, such as the Bedside index of severity in acute pancreatitis (BISAP), have only moderate diagnostic accuracy in the prediction of SAP (Mounzer et al., 2012). More recently, attention has also focused on assessing the association between SAP and individual laboratory parameters, such as admission albumin, blood urea nitrogen (BUN), high-density lipoprotein cholesterol level, and total cholesterol level on admission (Koutroumpakis et al., 2015; Hong et al., 2017b; Hong et al., 2017c; Hong et al., 2020).

Repeating serum amylase tests is thought to have no value in assessing the clinical progress of the patients or the ultimate prognosis once the diagnosis of AP has been made (Yadav et al., 2002). However, recently, Kumaravel et al. suggest a 10% decrease in the percentage of amylase during the first 2 days after admission was associated with a significantly decreased odds of SAP (Odds ratio, 0.93, 95% CI 0.87–0.98) (Kumaravel et al., 2015). However, it is not clear whether an increase in amylase from day 1 to day 2 would be associated with an increase in the incidence of SAP. On the other hand, the amylase increase is usually first detected 2–12 h after the onset of symptoms in AP. The level then peaks at 12–72 h and usually normalizes within 5 days (Frank and Gottlieb, 1999; Hong et al., 2017a). In clinical practice, serum amylase may increase or decrease during the first 2 days after admission. In our opinion, evaluation of the amylase day 2/amylase day 1 ratio may be more appropriate and comprehensive rather than that of an increase or decrease of amylase from day 1 to day 2 after admission as a potential predictor of severe acute pancreatitis. In addition, Fallah et al. suggested that nonlinear modeling procedures can prevent model misspecification and can provide information between prognostic factors and disease risk that is not revealed by the use of standard modeling techniques (Fallah et al., 2009). To the best of our knowledge, the non-linear correlation between the amylase day 2/amylase day 1 ratio and SAP has not been evaluated in the literature. Therefore, the current study aimed to assess the relationship between the amylase day 2/amylase day 1 ratio and SAP.

## Methods

### Inclusion and exclusion criteria

Patients with AP admitted to our hospital within 72 h of the onset of symptoms from 1 January 2012 to 31 December 2015 were retrospectively enrolled in the study. The AP diagnosis was based on the presence of two of the three features (pancreatic pain, amylase, and/or lipase ≥three times the upper limit of normal and characteristic findings on abdominal imaging) (Hong et al., 2011). The disease severity was stratified into mild, moderately severe, and severe according to the revised Atlanta classification (Banks et al., 2013). SAP consists of persistent organ failure (at least one of the three organs involved: cardiovascular failure, respiratory failure, and renal failure) for more than 48 h (Hong et al., 2017c). Exclusion criteria were: previous pancreatic surgery, pancreatitis due to endoscopic retrograde cholangiopancreatography (ERCP) or trauma, chronic pancreatitis, pancreatic cancer, patients receiving surgery or therapeutic ERCP during hospitalization, chronic renal disease, previous albuminuria, hepatitis, liver cirrhosis, and incomplete data records.

### Data collection

Age, gender, body mass index (BMI), and time from symptom onset to patient admission were recorded within 12 h of hospitalization. Serum amylase was measured on admission (day 1) and 24 h later (day 2) (Banks et al., 2013; Hong et al., 2017a). The amylase ratio was calculated as amylase day 2/amylase day 1. In addition, the BISAP score was calculated according to the laboratory and clinical data (Wu et al., 2008).

This study protocol was approved by the Ethics Committee of our hospital (date: 18-10-2016; number: 2016-211). This study was performed according to the principles expressed in the Declaration of Helsinki and written informed consent was obtained from all the subjects.

### Sample size

The sample size was calculated based on the identification of an independent dichotomous predictor in multivariable logistic regression analysis for SAP (Hong et al., 2020). With an α risk of 0.05 and β risk of 0.1, the prevalence of SAP was estimated to be 10% with a bilateral test, assuming a low correlation between the predictor and other covariates (R2 = 0.20). A sample of 309 patients was predicted to provide 80% power of detecting of an adjusted odds ratio (OR) of 3.0 for a dichotomous predictor with an overall prevalence of 70%.

### Statistical analysis

Categorical values were described by count and proportions and compared by Pearson’s χ^2^ test or Fisher’s exact test if there were few observations. A Shapiro–Wilk test was used to evaluate whether the continuous data had a normal distribution (Hong et al., 2017b). According to the results of the Shapiro–Wilk test, continuous values were expressed using mean ± standard deviation (SD), or median and interquartile range (IQR). Continuous data were compared using Student’s t-test or one-way analysis of variance if normality and homogeneity of variance. Conversely, the nonparametric Mann–Whitney test or Kruskal–Wallis non-parametric test was used if there was no normality and homogeneity of variance for continuous data (Hong et al., 2020). Nonlinearity in the relationship between the amylase ratio and SAP was assessed by univariable logistic regression with restricted cubic spline analysis (Hong et al., 2020). We used the default (5 knots) number of knots when performing restricted cubic spline analysis (knot points for amylase ratio levels: 0.096, 0.261, 0.432, 0.681, and 1.445). The cutoff of amylase ratio levels used to differentiate SAP from non-SAP was determined according to expected incidences of SAP predicted by restricted cubic spline analysis (Hong et al., 2020). Multivariable logistic analysis was also used to evaluate the relationship between the amylase ratio and SAP adjusted for potential confounders. We used our clinical experience, knowledge and previous study to select possible confounders for their potential association with amylase levels as follows: age, gender, body mass index, biliary etiology, and time interval before admission (Hong et al., 2017a). Odds ratios (OR) were calculated with 95% confidence intervals (CI). In order to evaluate the clinical usefulness of amylase ratio as an early predictor of SAP, the area under the receiver operating characteristic (ROC) curve (AUC) was used to evaluate the performance of predictions. Differences were assessed as being relevant when the two-tailed P-value <0.05 was reached.

## Results

### Clinical characteristics

A total of 464 patients, of whom 281 (60.6%) were male with a median age of 48 (37–63) years old, were included in the study (**Table 1**). The interval between the onset and admission was 1.8 ± 0.8 days. There was no significant difference with respect to the interval between the onset and admission among patients with different severity of disease (P = 0.73). It was 1.8 ± 0.8 days, 1.9 ± 0.8 days, and 1.9 ± 0.8 days for patients with mild, moderate severe, and severe AP, respectively. The median amylase levels on day 1 and day 2 were 743 (IQR 273–1,660) IU/L and 268 (IQR 119.5–587) IU/L, respectively. The most common cause of AP was the involvement of the biliary system (42.5%). Of all 464 patients, 348 (75.0%), 66 (14.2%), and 50 (10.8%) patients developed mild, moderately severe, and severe AP, respectively. Eight (1.72%) died during hospitalization. Patients with SAP had higher median serum amylase levels (941 IU/L, IQR 536–2,098 IU/L) on admission (day 1) compared to patients without SAP (666 IU/L, IQR 262–1,619 IU/L) (P = 0.0237).

**Table 1:** Demographic and Clinical Characteristics of 464 patients.

Characteristic	Value
Median age, years	48 (37–63)
Male sex, N (%)	281 (60.6%)
Time from pain onset to admission, days	1.8 ± 0.8
Mean BMI	23.5 (21.2–26.0)
Etiology	
Biliary, N (%)	197 (42.5)
Alcohol, N (%)	68 (14.7)
Hypertriglyceridemia, N (%)	27 (5.8)
Idiopathic, N (%)	157 (33.8)
Other, N (%)	15 (3.2)
Amylase at day 1 (IU/L)	743 (273–1,660)
Amylase at day 2 (IU/L)	268 (119.5–587)
Hematocrit	0.43 (0.38–0.46)
BUN, mmol/L	4.8 (3.7–6.4)
Outcomes	
Severity of acute pancreatitis	
Mild, N (%)	348 (75)
Moderately severe, N (%)	66 (14.2)
Severe, N (%)	50 (10.8)
Mean hospital days	10 (7–14)
Persistent organ failure N (%)	50 (10.8)
Death, N (%)	8 (1.72)

Data are shown either as number of observations, mean ± standard deviation (SD), percentage, median or interquartile range (IQR).

### Non-linear correlation between amylase day 2 to day 1 ratio and incidence of SAP

**Figure 1:**
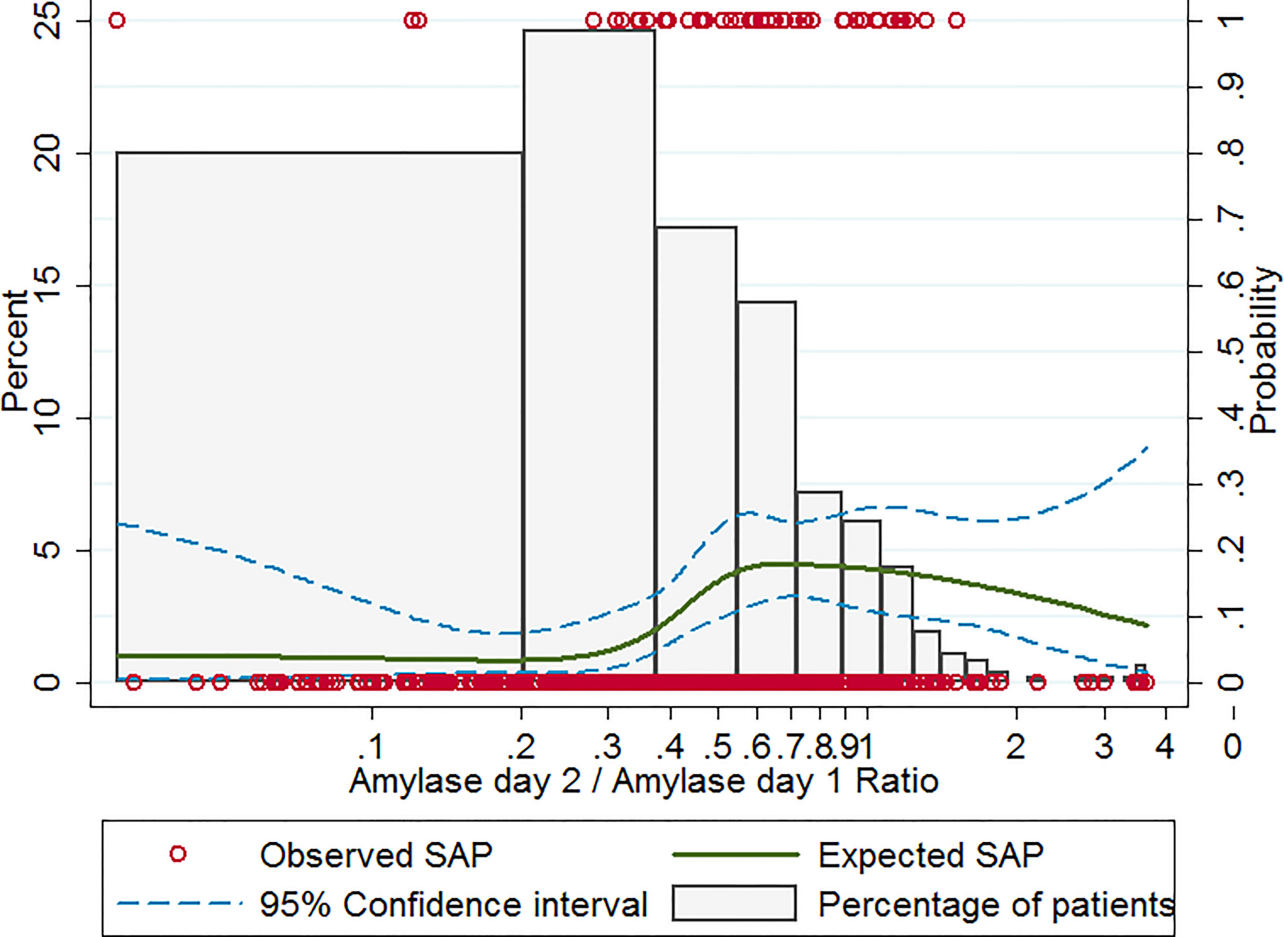
Observed and expected incidence of SAP in patients with different amylase ratio levels. Histograms show the percent distribution of study patients at different amylase ratio levels (the left-hand y-axis). The red circles give the observed incidence of SAP for each patient (the right-hand y-axis). The solid curve gives the expected incidence of SAP based on restricted cubic spline analysis (the right-hand y-axis). The dashed curves represent the 95% confidence intervals for the expected incidence of SAP (the right-hand y-axis). SAP, severe acute pancreatitis.

Based on univariable logistic regression with restricted cubic spline analysis, a non-linear association between the amylase ratio and SAP was observed (**Figure 1**). In patients with an amylase ratio of <0.3, the expected incidence of SAP was low and did not significantly change with amylase ratio. While in patients with an amylase ratio of ≥0.3, the expected incidence of SAP increased rapidly with amylase ratio and reached a peak in patients with an amylase ratio of 0.6. Therefore, 0.3 was used as the cutoff of the amylase ratio to divide patients into different study groups.

### Amylase ratio was independently associated with SAP

**Figure 2:**
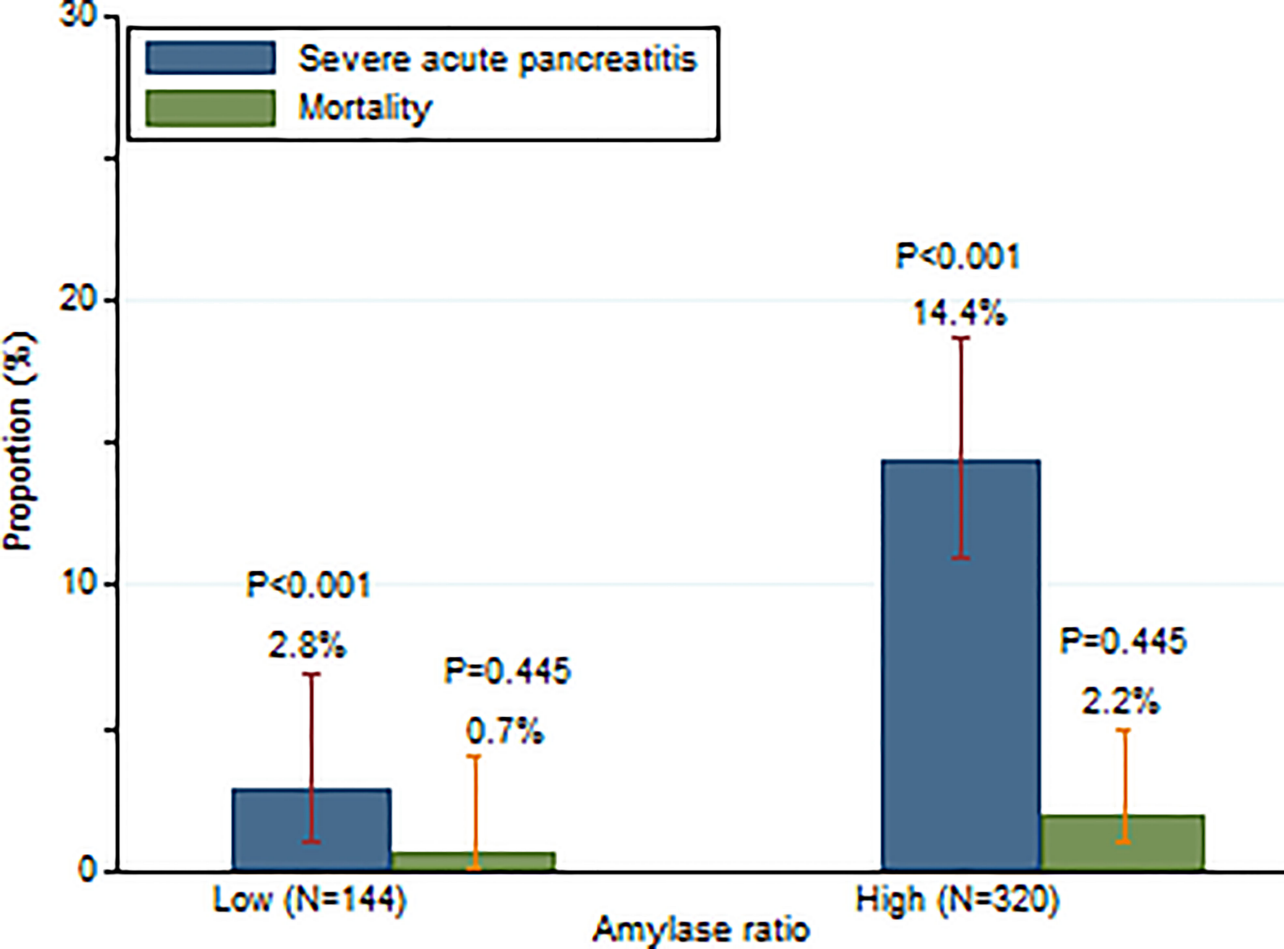
Incidence of SAP and mortality in patients with high and low serum amylase day 2/amylase day 1 ratios.

When 0.3 was used as the cut-off of amylase ratio, as shown in **Figure 2**, 14.4% (46/320) patients developed SAP with a high amylase ratio (≥0.3) as compared to 2.8% (4/144) with a low amylase ratio (<0.3) (P <0.001). Similarly, patients with a high amylase ratio (≥0.3) had a trend towards higher mortality (7/320, 2.2%) than patients with a low amylase ratio (<0.3) (1/144, 0.7%) during hospitalization, although it did not reach statistical significance (P = 0.445) **(** **Figure 2** **)**.

Multivariable logistic regression indicated that a high amylase ratio (≥0.3) (OR: 6.62; 95% CI: 2.27–19.36; P = 0.001) was independently associated with the development of SAP after adjustment for age, gender, BMI, biliary etiology, and time from pain onset to admission.

### Amylase ratio as predictor of SAP

**Figure 3:**
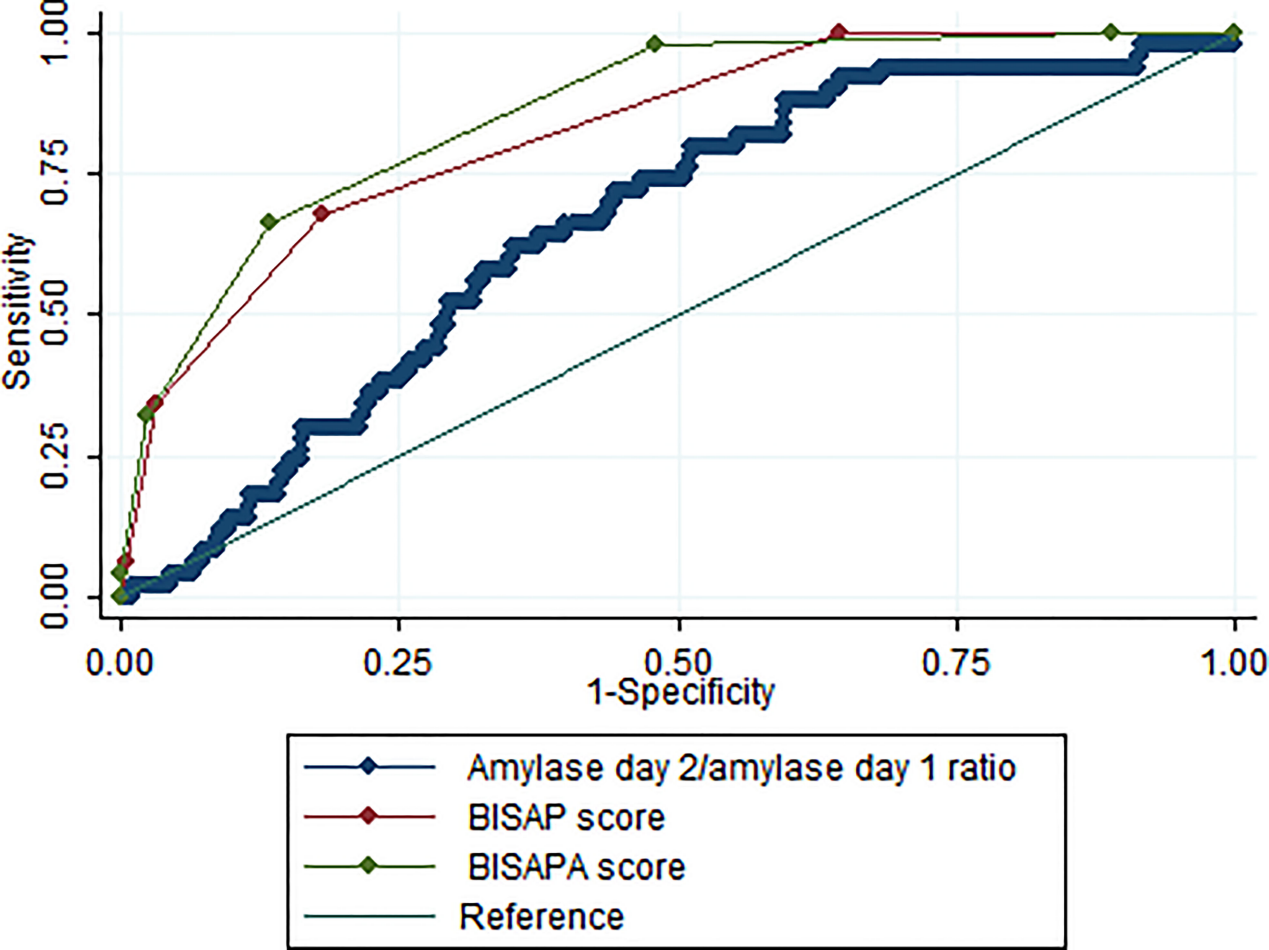
The ROC curves for serum amylase day 2/amylase day 1 ratio, BISAP score, and BISAPA score for SAP prediction. BISAP, bedside index of severity in AP; SAP, severe acute pancreatitis; BISAPA, bedside index of severity in acute pancreatitis and amylase day 2/amylase day 1 ratio.

Based on ROC analysis, the AUC for the amylase ratio for the prediction of SAP was 0.65 ± 0.04 (**Figure 3**). With a cut-off value of 0.3, the amylase ratio achieved a sensitivity of 92.0% and a specificity of 33.8%. Overall, the diagnostic performance of the amylase ratio was inferior to the BISAP score (AUC = 0.83 ± 0.03) (**Figure 3**). When amylase ratio ≥0.3 was counted as 1 point and added to the BISAP score to build a new model named the BISAPA (BISAP plus Amylase ratio) score (AUC = 0.86 ± 0.02), it significantly improved diagnostic power compared to the original BISAP score (**Figure 3**). With a cut-off value of 3, the BISAPA score achieved a sensitivity of 66.0%, a specificity of 86.7%, and diagnostic accuracy of 84.48%.

## Discussion

Although amylase is useful in the diagnosis of pancreatitis, it correlates poorly with the severity of the illness (Yadav et al., 2002). As a result, the level of elevation of amylase is not included in the major tools used to assess the severity of illness, such as Ranson’s criteria and BISAP score. The American College of Gastroenterology guidelines states that daily measurement of amylase after the initial diagnosis has limited value in assessing the clinical progress of the illness or ultimate prognosis (Tenner et al., 2013). However, recently, Kumaravel et al. (2015) suggested that the percentage change in amylase from admission to day 2b (which was calculated as ([amylase day 1 − amylase day2]/amylase day 1)) was associated with the severity of AP (Kumaravel et al., 2015). However, serum amylase may increase or decrease during the first 2 days after admission. Therefore, the amylase ratio, in clinical practice, which was calculated as (amylase day 2/amylase day 1), might be more suitable than the percentage change in amylase as a potential predictor of SAP.

Our study suggested that patients with a high amylase day 2/day 1 ratio (≥0.3) had a higher incidence of SAP than patients with a low amylase day 2/day 1 ratio (<0.3) (14.4% vs. 2.8%, P <0.001) **(** **Figure 2** **)**. These results indicate that 97.2% of patients would not develop SAP if their amylase on day 2 decreased by 70% compared to amylase on day 1. However, 14.4% of patients would develop SAP if their amylase on day 2 either decreased by less than 70% compared to day 1 or increased. Multivariable logistic regression indicated that a high amylase ratio (≥0.3) (OR: 6.62; 95% CI: 2.27–19.36; P = 0.001) was independently associated with the development of SAP after adjusting age, gender, BMI, biliary etiology, and time from pain onset to admission.

Based on ROC analysis, the AUC for the amylase ratio for the prediction of SAP was 0.65 (95% CI: 0.58–0.73) (**Figure 3**), which means that a serum amylase day 2/amylase day 1 ratio had moderate diagnostic accuracy for the prediction of SAP. With a cut-off of 0.3, the serum amylase day 2/amylase day 1 ratio achieved a sensitivity of 92.0% and specificity of 33.8%. Therefore, patients with a serum amylase day 2/amylase day 1 ratio of >0.3 should be transferred to an intensive care unit setting. The advantages of amylase ratio estimation are its technical simplicity, easy availability, and high sensitivity. However, its disadvantage is its low specificity with this cut-off value. The diagnostic performance of the amylase ratio for the prediction of SAP was significantly inferior to the BISAP score (AUC: 0.65 vs.0.83; P <0.001) **(** **Figure 3** **)**. Therefore, the serum amylase day 2/amylase day 1 ratio may be used as an additional supplement tool but not a substitute when compared to BISAP score. When amylase ratio ≥0.3 was counted as 1 point and added to the BISAP score to build a new model named the BISAPA score (AUC = 0.86), it significantly improved diagnostic power compared to the original BISAP score (AUC = 0.83) (**Figure 3**). With a cut-off value of 3, the BISAPA score achieved a sensitivity of 66.0%, specificity of 86.7%, and diagnostic accuracy of 84.48%.

One of the novelties of our study is that we fully investigated the relationship between amylase day 2/amylase day 1 ratio and SAP and determined the best cut-off value of serum amylase day 2/amylase day 1 ratio for the prediction of SAP by using a nonlinear model (restricted cubic spline analysis) (**Figure 1**). The restricted cubic spline model can transform an independent continuous variable and analyze the nonlinear effects of an independent variable on disease severity (Desquilbet and Mariotti, 2010). It generally provides a better fit to the data and also has the effect of reducing the degrees of freedom (Desquilbet and Mariotti, 2010). The other novelty is that we developed a BISAPA score, which incorporated both the serum amylase day 2/amylase day 1 ratio and the BISAP score. It could help us better stratify the severity of AP and predict outcomes in AP, as well as achieve high diagnostic accuracy. Early staging of SAP is critical to enable adequate triage of these patients to the intensive care unit and a timely treatment plan (Dar et al., 2021). Recently, Choi et al. proposed that serum phosphate level after ERCP can be used as a reliable prognostic marker in predicting the severity of post-ERCP pancreatitis (Choi et al., 2018). Agarwala et al. identified that gastrointestinal failure is an independent predictor of mortality in patients with AP (Agarwala et al., 2020). Trikudanathan et al. suggested that decreased skeletal muscle density was independently associated with in-hospital mortality in necrotizing pancreatitis patients and can be usefully incorporated into computed tomography-based predictive scoring models as a prognostic marker (Trikudanathan et al., 2021). Liu et al. suggested that the volume and mean computed tomography density of necrosis based on contrast-enhanced computed tomography can provide early prediction of organ failure and the need for intervention in patients with acute necrotizing pancreatitis (Liu et al., 2021). It will be necessary and interesting to compare the performance of our BISAPA score with the aforesaid indexes in predicting the disease severity of AP in the future.

Nevertheless, our study has some limitations. First, the important limitation is that serum lipase on day 1 and day 2 was not tested in most patients in our study. Therefore, we did not investigate the relationship between SAP and lipase. As a result, our results may not be generalized to some countries, such as Germany, where amylase measurements have largely been substituted for lipase measurements. However, according to the current international consensus (Banks et al., 2013), an increase in amylase activity is still one of the primary criteria for the diagnosis of AP. Amylase testing other than lipase is still routinely performed at many healthcare institutions in clinical practice in most countries (such as mainland China in the world), although lipase is believed to offer superior sensitivity and specificity compared to amylase for the diagnosis of AP (Hong et al., 2017a). In addition, Chase et al. suggested that checking for lipase and amylase simultaneously does not result in improved diagnostic accuracy because amylase and lipase are closely correlated (Chase et al., 1996). Second, not all patients had records of C-reactive protein, serum lipase, and disease severity scores such as Acute Physiology and Chronic Health Evaluation (APACHE) II due to the retrospective study design. This made it difficult for us to further analyze the relationship between the amylase day 2 to day 1 ratio and these indexes. It will be necessary and interesting to evaluate these correlations in the future. Third, in AP, the levels of amylase usually peak at 48 h. Over the period of the next 5–7 days after onset, amylase levels typically tend to normalize (Hong et al., 2017a). Only patients who were admitted to the hospital within 72 h of the onset of symptoms were enrolled in our study. Therefore, our results may not be generalized to patients with a delay in time from pain onset to admission. Additionally, previous studies suggest that hospital volume influences the clinical outcome in both patients with mild and those with SAP (Singla et al., 2009; Murata et al., 2011). In our study, there were very few patients referred from other hospitals. Therefore, the results from our hospital may not be generalized to other hospitals with a high proportion of interhospital transfers. Last, similar to the change in amylase and body mass index (CAB) score (Kumaravel et al., 2015; Zheng et al., 2019), the new Japanese severity score (Ueda et al., 2009) and the modified Glasgow score (Mounzer et al., 2012), our amylase day 2 to day 1 ratio also requires 48 h to complete, which means it cannot be used at bedside as a predictor of SAP within 24 h of admission in clinical practice. However, AP is a dynamic and evolving process that involves multiple systems and the risk of organ complications (Papachristou et al., 2010). Therefore, the amylase day 2 to day 1 ratio could still be useful on day 2 after admission.

## Conclusion

In conclusion, there is a non-linear correlation between the amylase day 2/amylase day 1 ratio and SAP incidence. The serum amylase day 2/amylase day 1 ratio represents an additional tool that is easy to perform and could serve as a cheap marker to stratify patients at risk of SAP without any need for complex calculations. When the amylase ratio of ≥0.3 was counted as 1 point and added to the BISAP score to build a new model named the BISAPA score, it significantly improved diagnostic power compared to the original BISAP score.

## Data availability statement

The raw data supporting the conclusions of this article will be made available by the authors, without undue reservation.

## Ethics statement

This study protocol was approved by the Ethics Committee of our hospital (date: 2016-10-18; number: 2016-211). The patients/participants provided their written informed consent to participate in this study.

## Author contributions

WH conceived the study and carried out majority of the work. WH and LZ participated in data collection and conducted data analysis. WH, YL, and MQ drafted and revised the manuscript. YY, ZB, MZ, VZ, and WG helped to finalize the manuscript. All authors listed have made a substantial, direct, and intellectual contribution to the work and approved it for publication.

## Funding

This work was supported by the Zhejiang Medical and Health Science and Technology Plan Project (Number: 2022KY886) and the Wenzhou Science and Technology Bureau (Number: Y2020010).

## Conflict of interest

The authors declare that the research was conducted in the absence of any commercial or financial relationships that could be construed as a potential conflict of interest.

## Publisher’s note

All claims expressed in this article are solely those of the authors and do not necessarily represent those of their affiliated organizations, or those of the publisher, the editors and the reviewers. Any product that may be evaluated in this article, or claim that may be made by its manufacturer, is not guaranteed or endorsed by the publisher.

## Abbreviations

AUC, area under the receiver operating characteristic curve; BISAP, Bedside index of severity in acute pancreatitis; BMI, body mass index; BUN, blood urea nitrogen; CI, confidence interval; ERCP, endoscopic retrograde cholangiopancreatography; HRS, hours; IQR, interquartile range; OR, odds ratio; ROC, receiver operating characteristic curve; SAP, severe acute pancreatitis; SD, standard deviation.

## References

[B1] Agarwala R. Rana S. S. Sharma R. Kang M. Gorsi U. Gupta R. (2020). Gastrointestinal failure is a predictor of poor outcome in patients with acute pancreatitis. Dig. Dis. Sci. 65, 2419–2426. doi: 10.1007/s10620-019-05952-5 31722056

[B2] Banks P. A. Bollen T. L. Dervenis C. Gooszen H. G. Johnson C. D. Sarr M. G.. (2013). Classification of acute pancreatitis--2012: Revision of the Atlanta classification and definitions by international consensus. Gut 62, 102–111. doi: 10.1136/gutjnl-2012-302779 23100216

[B3] Chase C. W. Barker D. E. Russell W. L. Burns R. P. (1996). Serum amylase and lipase in the evaluation of acute abdominal pain. Am. Surg. 62, 1028–1033. Available at: https://pubmed.ncbi.nlm.nih.gov/8955242/ 8955242

[B4] Choi Y. H. Jang D. K. Lee S. H. Jang S. Choi J. H. Kang J.. (2018). Utility of serum phosphate as a marker for predicting the severity of post-endoscopic retrograde cholangiopancreatography pancreatitis. United Eur. Gastroenterol. J. 6, 895–901. doi: 10.1177/2050640618764168 PMC6047292 30023067

[B5] Dar G. Goldberg S. N. Hiller N. Caplan N. Sosna J. Appelbaum L.. (2021). CT severity indices derived from low monoenergetic images at dual-energy CT may improve prediction of outcome in acute pancreatitis. Eur. Radiol. 31, 4710–4719. doi: 10.1007/s00330-020-07477-2 33404695

[B6] Desquilbet L. Mariotti F. (2010). Dose-response analyses using restricted cubic spline functions in public health research. Stat. Med. 29, 1037–1057. doi: 10.1002/sim.3841 20087875

[B7] Fallah N. Mohammad K. Nourijelyani K. Eshraghian M. R. Seyyedsalehi S. A. Raiessi M.. (2009). Nonlinear association between serum testosterone levels and coronary artery disease in Iranian men. Eur. J. Epidemiol. 24, 297–306. doi: 10.1007/s10654-009-9336-9 19357974

[B8] Frank B. Gottlieb K. (1999). Amylase normal, lipase elevated: is it pancreatitis? a case series and review of the literature. Am. J. Gastroenterol. 94, 463–469. doi: 10.1111/j.1572-0241.1999.878_g.x 10022647

[B9] Hong W. Dong L. Huang Q. Wu W. Wu J. Wang Y. (2011). Prediction of severe acute pancreatitis using classification and regression tree analysis. Dig. Dis. Sci. 56, 3664–3671. doi: 10.1007/s10620-011-1849-x 21833749

[B10] Hong W. Geng W. Chen B. Basharat Z. Wu Q. Zimmer V.. (2017a). Predictors of acute pancreatitis with low elevation of serum amylase. Ther. Clin. Risk Manag. 13, 1577–1584. doi: 10.2147/TCRM.S147594 29276389 PMC5734230

[B11] Hong W. Lillemoe K. D. Pan S. Zimmer V. Kontopantelis E. Stock S.. (2019). Development and validation of a risk prediction score for severe acute pancreatitis. J. Transl. Med. 17, 146. doi: 10.1186/s12967-019-1903-6 31068202 PMC6505180

[B12] Hong W. Lin S. Zippi M. Geng W. Stock S. Basharat Z.. (2017b). Serum albumin is independently associated with persistent organ failure in acute pancreatitis. Can. J. Gastroenterol. Hepatol. 2017, 10. doi: 10.1155/2017/5297143 PMC5632885 29147647

[B13] Hong W. Lin S. Zippi M. Geng W. Stock S. Zimmer V.. (2017c). High-density lipoprotein cholesterol, blood urea nitrogen, and serum creatinine can predict severe acute pancreatitis. BioMed. Res. Int. 2017, 1648385. doi: 10.1155/2017/1648385 28904946 PMC5585681

[B14] Hong W. Zimmer V. Basharat Z. Zippi M. Stock S. Geng W.. (2020). Association of total cholesterol with severe acute pancreatitis: A U-shaped relationship. Clin. Nutr. 39, 250–257. doi: 10.1016/j.clnu.2019.01.022 30772093

[B15] Koutroumpakis E. Wu B. U. Bakker O. J. Dudekula A. Singh V. K. Besselink M. G.. (2015). Admission hematocrit and rise in blood urea nitrogen at 24 h outperform other laboratory markers in predicting persistent organ failure and pancreatic necrosis in acute pancreatitis: A post hoc analysis of three Large prospective databases. Am. J. Gastroenterol. 110, 1707–1716. doi: 10.1038/ajg.2015.370 26553208

[B16] Kumaravel A. Stevens T. Papachristou G. I. Muddana V. Bhatt A. Lee P. J.. (2015). A model to predict the severity of acute pancreatitis based on serum level of amylase and body mass index. Clin. Gastroenterol. Hepatol. 13, 1496–1501. doi: 10.1016/j.cgh.2015.03.018 25818080

[B17] Liu N. He J. Hu X. Xu S. F. Su W. Luo J. F.. (2021). Acute necrotising pancreatitis: measurements of necrosis volume and mean CT attenuation help early prediction of organ failure and need for intervention. Eur. Radiol. 31, 7705–7714. doi: 10.1007/s00330-021-07840-x 33758956

[B18] Mounzer R. Langmead C. J. Wu B. U. Evans A. C. Bishehsari F. Muddana V.. (2012). Comparison of existing clinical scoring systems to predict persistent organ failure in patients with acute pancreatitis. Gastroenterology 142, 1476–1482; quiz e1415-1476. doi: 10.1053/j.gastro.2012.03.005 22425589

[B19] Murata A. Matsuda S. Mayumi T. Yokoe M. Kuwabara K. Ichimiya Y.. (2011). Effect of hospital volume on clinical outcome in patients with acute pancreatitis, based on a national administrative database. Pancreas 40, 1018–1023. doi: 10.1097/MPA.0b013e31821bd233 21926541

[B20] Papachristou G. I. Muddana V. Yadav D. O'connell M. Sanders M. K. Slivka A.. (2010). Comparison of BISAP, ranson's, APACHE-II, and CTSI scores in predicting organ failure, complications, and mortality in acute pancreatitis. Am. J. Gastroenterol. 105, 435–441; quiz 442. doi: 10.1038/ajg.2009.622 19861954

[B21] Singla A. Simons J. Li Y. Csikesz N. G. Ng S. C. Tseng J. F.. (2009). Admission volume determines outcome for patients with acute pancreatitis. Gastroenterology 137, 1995–2001. doi: 10.1053/j.gastro.2009.08.056 19733570

[B22] Tenner S. Baillie J. Dewitt J. Vege S. S. American College Of, G. (2013). American College of gastroenterology guideline: Management of acute pancreatitis. Am. J. Gastroenterol. 108, 1400–1415; 1416. doi: 10.1038/ajg.2013.218 23896955

[B23] Trikudanathan G. Vantanasiri K. Faizi N. Munigala S. Vanek P. Schat R.. (2021). Decreased skeletal muscle density is an independent predictor of mortality in necrotizing pancreatitis- a single tertiary center experience in 507 patients. Pancreatology S1424-3903(21)00160-5. doi: 10.1016/j.pan.2021.05.010 34020888

[B24] Ueda T. Takeyama Y. Yasuda T. Kamei K. Satoi S. Sawa H.. (2009). Utility of the new Japanese severity score and indications for special therapies in acute pancreatitis. J. Gastroenterol. 44, 453–459. doi: 10.1007/s00535-009-0026-x 19308309

[B25] Wu B. U. Johannes R. S. Sun X. Tabak Y. Conwell D. L. Banks P. A. (2008). The early prediction of mortality in acute pancreatitis: A large population-based study. Gut 57, 1698–1703. doi: 10.1136/gut.2008.152702 18519429

[B26] Yadav D. Agarwal N. Pitchumoni C. S. (2002). A critical evaluation of laboratory tests in acute pancreatitis. Am. J. Gastroenterol. 97, 1309–1318. doi: 10.1111/j.1572-0241.2002.05766.x 12094843

[B27] Zheng L. Hong W. Geng W. Stock S. Pan J. (2019). A comparison of the BISAP score and amylase and BMI (CAB) score versus for predicting severe acute pancreatitis. Acta Gastroenterol. Belg. 82, 397–400. Available at: https://pubmed.ncbi.nlm.nih.gov/31566327/ 31566327

